# Cold atmospheric microwave plasma (CAMP) stimulates dermal papilla cell proliferation by inducing β-catenin signaling

**DOI:** 10.1038/s41598-023-30122-z

**Published:** 2023-02-22

**Authors:** Kuljira Mongkolpobsin, Chanin Sillapachaiyaporn, Pattawika Lertpatipanpong, Kanokkan Boonruang, Cheol-Yong Hwang, Tewin Tencomnao, Seung Joon Baek

**Affiliations:** 1 Laboratory of Signal Transduction, Research Institute for Veterinary Science, College of Veterinary Medicine, Seoul National University, 1 Gwanak-Ro, Gwanak-Gu, Seoul, 08826 Korea; 2 Program in Clinical Biochemistry and Molecular Medicine, Department of Clinical Chemistry, Faculty of Allied Health Sciences, Chulalongkorn University, Bangkok, 10330 Thailand; 3 Laboratory of Veterinary Dermatology, Research Institute for Veterinary Science, College of Veterinary Medicine, Seoul National University, Seoul, 08826 Korea

**Keywords:** Biochemistry, Biotechnology, Molecular biology, Medical research, Energy science and technology

## Abstract

Hair loss or alopecia is an unpleasant symptom that exacerbates an individual's self-esteem and requires appropriate treatment. The Wnt/β-catenin signaling is a central pathway that promotes dermal papilla induction and keratinocyte proliferation during hair follicle renewal. GSK-3β inactivated by its upstream Akt and ubiquitin-specific protease 47 (USP47) has been shown to inhibit β-catenin degradation. The cold atmospheric microwave plasma (CAMP) is microwave energy enriched with mixtures of radicals. CAMP has been reported to have antibacterial and antifungal activities with wound healing activity against skin infection; however, the effect of CAMP on hair loss treatment has not been reported. We aimed to investigate the effect of CAMP on promoting hair renewal in vitro and to elucidate the molecular mechanism, targeting β-catenin signaling and YAP/TAZ, the co-activators in the Hippo pathway, in human dermal papilla cells (hDPCs). We also evaluated plasma effects on the interaction between hDPCs and HaCaT keratinocytes. The hDPCs were treated with plasma-activating media (PAM) or gas-activating media (GAM). The biological outcomes were determined by MTT assay, qRT-PCR, western blot analysis, immunoprecipitation, and immunofluorescence. We found that β-catenin signaling and YAP/TAZ were significantly increased in PAM-treated hDPCs. PAM treatment also induced β-catenin translocation and inhibited β-catenin ubiquitination by activating Akt/GSK-3β signaling and upregulating USP47 expression. In addition, hDPCs were more aggregated with keratinocytes in PAM-treated cells compared with control. HaCaT cells cultured in a conditioned medium derived from PAM-treated hDPCs exhibited an enhancing effect on activating YAP/TAZ and β-catenin signaling. These findings suggested that CAMP may be a new therapeutic alternative for alopecic treatment.

## Introduction

Hair is served as an important structure that fulfills an individual's self-confidence. Although hair loss is not considered a severe disease, it can impair an overall appearance, lowering self-esteem and quality of life. However, the FDA-approved drugs, minoxidil and finasteride, require repeated use for effective treatment, which has been shown to induce adverse effects^1,2^. Therefore, finding effective and alternative hair loss therapy is a key topic that needs further research.

The cold atmospheric plasma (CAP) is an ionized gas that contains mixtures of reactive oxygen species, reactive nitrogen species, ultraviolet (UV), and photons. CAP is ignited from a high-voltage electrode, and plasma is delivered to the target sites through the carrier gas at a low temperature. The plasma level can be controlled by adjusting the power consumption and gas velocity^3^. The CAP has further developed into a cold atmospheric microwave plasma (CAMP) to generate a high density of radicals in the atmospheric condition. Compared to other conventional CAP, CAMP has several benefits in biomedical fields. This CAMP generates mild heat, probably providing additional benefits for hair growth, and plasma density or reactive species concentration can reach further distance from the device.

Recent reports suggested that CAMP affects antifungal activity^4^, antibacterial activity^5^, anticancer activity^6^, and promotes stem cell proliferation^7^. Although CAP activates dermal papilla cell proliferation via Wnt/β-catenin signaling^8^, the effect of CAMP on stimulating hair growth has not been elucidated. In this research, we used the CAMP, a 2.45 GHz microwave energy containing a higher density of radicals than other types of CAP.

The hair follicle cycle consists of three phases; anagen, catagen, and telogen. Among these processes, the β-catenin signaling pathway is vital in promoting dermal papilla cell (DPCs) induction and keratinocyte division, which is responsible for maintaining the hair follicular compartment during hair regeneration^9^. The β-catenin is usually located in the cytoplasm and translocated into the nucleus after phosphorylation at the serine 552 position^10^. However, the phosphorylation at the serine 33, 37, or threonine 41 sites of β-catenin recruits ubiquitin to bind to itself, resulting in the degradation of β-catenin^11^. The activated β-catenin is a transcriptional activator of cell proliferation-related genes, cyclin D1, and c-Myc. During the anagen phase, GSK-3β is also phosphorylated to an inactive form by its upstream suppressor Akt. Since GSK-3β regulates β-catenin ubiquitination, the inactivation of GSK-3β results in β-catenin stabilization, which induces DPCs and keratinocytes proliferation^12^. A study also found that USP47, a deubiquitination protein, significantly inhibits β-catenin ubiquitination and promotes cell proliferation^13^.

In the bald area, hair follicles decrease in size, indicating a defective DPCs induction which contributes to an impairment of hair follicle renewal; however, restoring the β-catenin signaling pathway significantly induces hair follicle development^14,15^. Therefore, we aimed to study the effect of CAMP on enhancing Akt/β-catenin and USP47 signaling pathways in hDPCs. We hypothesized that CAMP promotes hDPCs aggregation in co-culture conditions between hDPCs and HaCaT keratinocytes. Since the epithelial-mesenchymal interactions are the main mechanism for initiating hair follicle formation, we also determined the effect of conditioned media (CM) derived from hDPCs on inducing CAMP-treated HaCaT keratinocyte proliferation.

## Results

### CAMP promotes the growth of human dermal papillary cells

**Figure 1:**
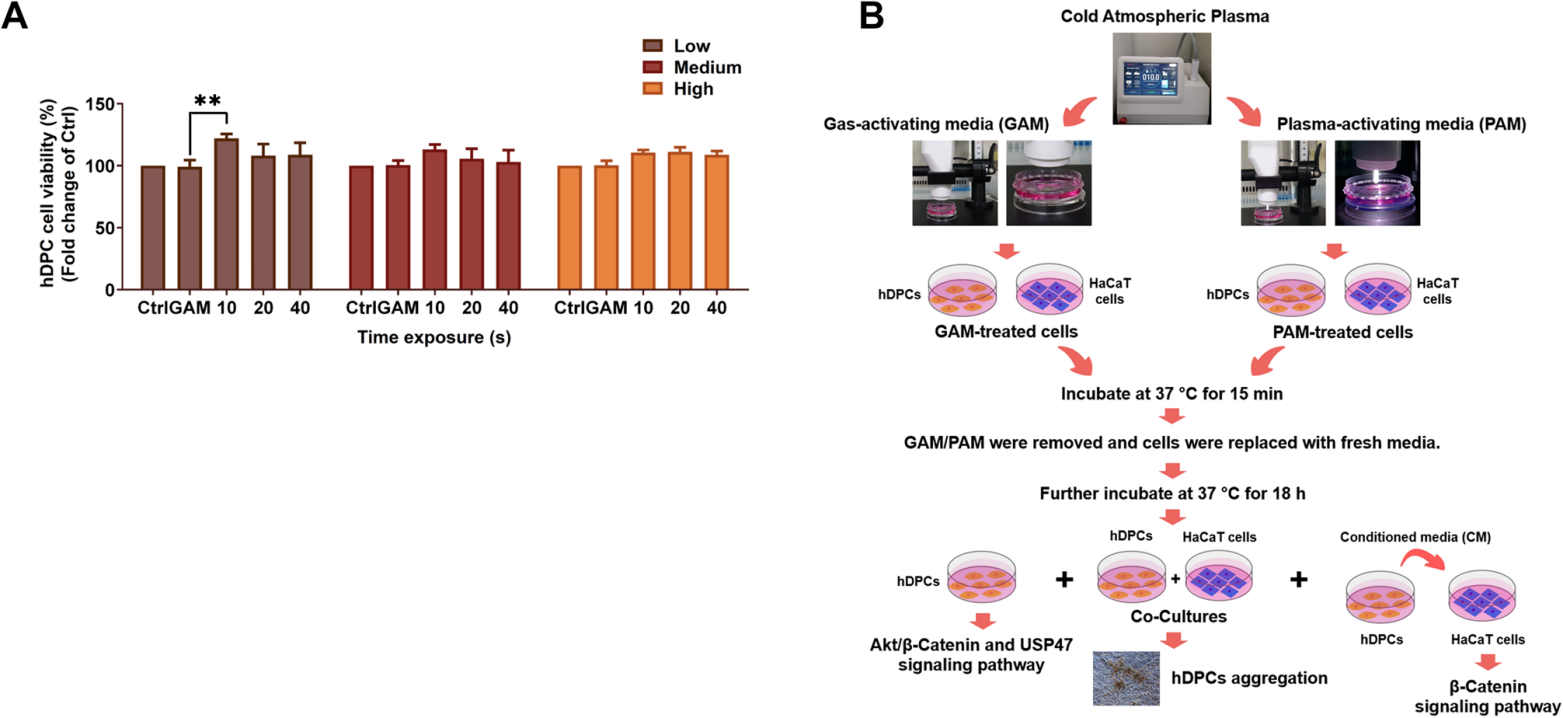
The CAMP promotes hDPC proliferation. (**A**) The cell viability of hDPCs against PAM. The hDPCs were grown for 15 min in the presence of PAM treated with CAMP at 30W (Low), 35W (Medium), and 40W (High) for 10, 20, and 40 s, respectively. The cells were replaced with fresh media and further grown for 18 h. MTT assay was performed as described in the method section. Ctrl represents non-treated hDPCs, whereas GAM indicates gas-activating media for 40 s. All data are shown as the mean ± SD of experiments conducted in triplicate. Statistical significance; **P < 0.01. (**B**) Schematic of CAMP treatment in hDPCs and HaCaT cells.

Several studies indicated that plasma exposure enhances hair growth^16^. To investigate the cell viability of PAM-treated cells, hDPCs were treated with different doses of PAM for 15 min and further incubated for 18 h after replacing media. Cell viability was evaluated by MTT assay. The results showed that PAM treatment significantly increases cell viability in hDPCs, compared with GAM control. In contrast, the other doses showed no significant difference (Fig. 1A). With this result, we established the protocol for elucidating the molecular mechanism by which CAMP affects hair growth (Fig. 1B). Hence, PAM for 10 s was selected for further experiments.

### PAM upregulates protein expression of the β-catenin signaling pathway and YAP/TAZ in hDPCs

**Figure 2:**
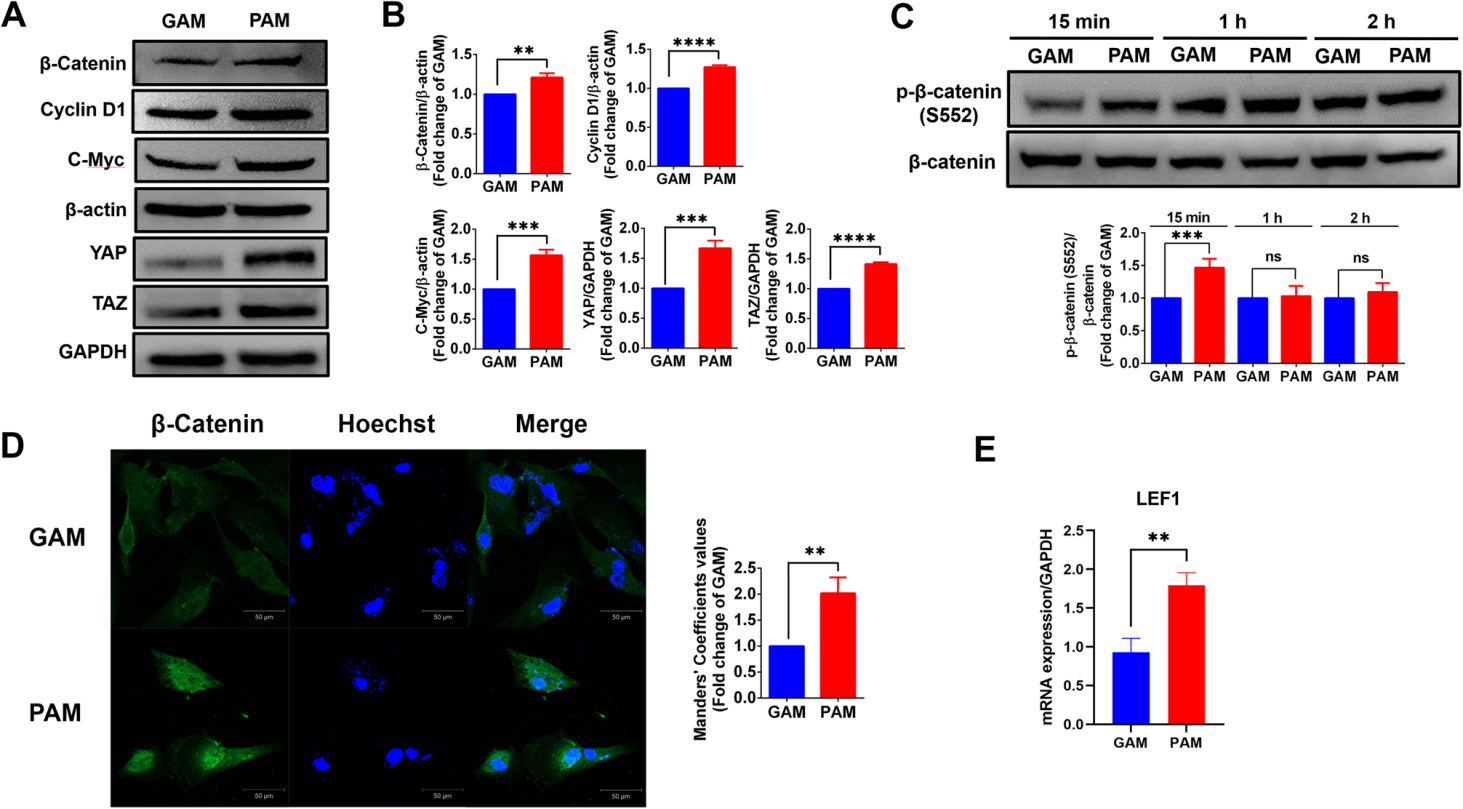
PAM upregulates protein expression of the β-catenin signaling pathway and YAP/TAZ and enhances β-catenin translocation in hDPCs. (**A**) Representative immunoblots and (**B**) Relative fold-change expressions of β-catenin, cyclin D1, c-Myc, YAP, and TAZ in PAM-treated hDPCs, after 18 h of incubation. (**C**) Representative immunoblots and relative fold-change expression of p-β-catenin (S552)/β-catenin level in PAM-treated hDPCs, compared to gas control, at 15 min, 1 h, and 2 h of incubation times. (**D**) Representative images of β-catenin immunofluorescence staining and quantitative nuclear β-catenin fluorescence intensity of GAM and PAM against hDPCs for 15 min incubation. The cells were observed under a confocal microscope with 20 × magnification. The scale bar indicates a 50 µm distance. (**E**) The mRNA expressions of LEF1 genes in PAM-treated hDPCs, after 18 h of incubation. All data are shown as the mean ± SD of experiments conducted in triplicate. Statistical significance; **P < 0.01; ***P < 0.001, and ****P < 0.0001. ns indicates not significant (*P* > 0.05).

Several studies indicate that the β-catenin signaling pathway is a significant contributor that controls dermal papilla cell proliferation and follicular keratinocyte differentiation. Its component proteins are important factors in hair growth^17^. Hence, we evaluated the effect of PAM-treated hDPCs on β-catenin expression and its target proteins by western blot analysis. The results indicated that the protein expressions of β-catenin, cyclin D1, and c-Myc were significantly increased in PAM-treated hDPCs, compared to GAM control (Fig. 2A,B). It has been reported that YAP and TAZ, the transcriptional co-activator proteins in the Hippo pathway, accumulate in the basal layer cells of hair follicles and participate in inducing hair regeneration^18^. Thus, we measured the protein expression of YAP and TAZ in the PAM-treated condition. The results showed that PAM-treated cells exhibited significant YAP and TAZ protein expression induction compared to GAM-treated cells (Fig. 2A,B). To evaluate β-catenin activity, we measured the expression level of phospho-β-catenin at S552, the specific site which regulates β-catenin translocation into the nucleus, and found that PAM-treated cells showed significant activation of β-catenin translocation (Fig. 2C). The results were confirmed by immunofluorescent assay. In the PAM-treated condition, β-catenin was more accumulated within the nucleus, compared to GAM-treated cells (Fig. 2D). In addition, since LEF1 is a co-transcriptional activator of β-catenin for inducing the expression of growth-promoting genes^19^, we next investigated the mRNA expression of LEF1. The result showed that PAM significantly upregulates LEF1 expression, compared to GAM (Fig. 2E). All these results suggested that PAM promotes to enhance β-catenin translocation and activates YAP/TAZ in the Hippo pathway in DPCs.

### PAM impeded β-catenin degradation via USP47 in hDPCs

**Figure 3:**
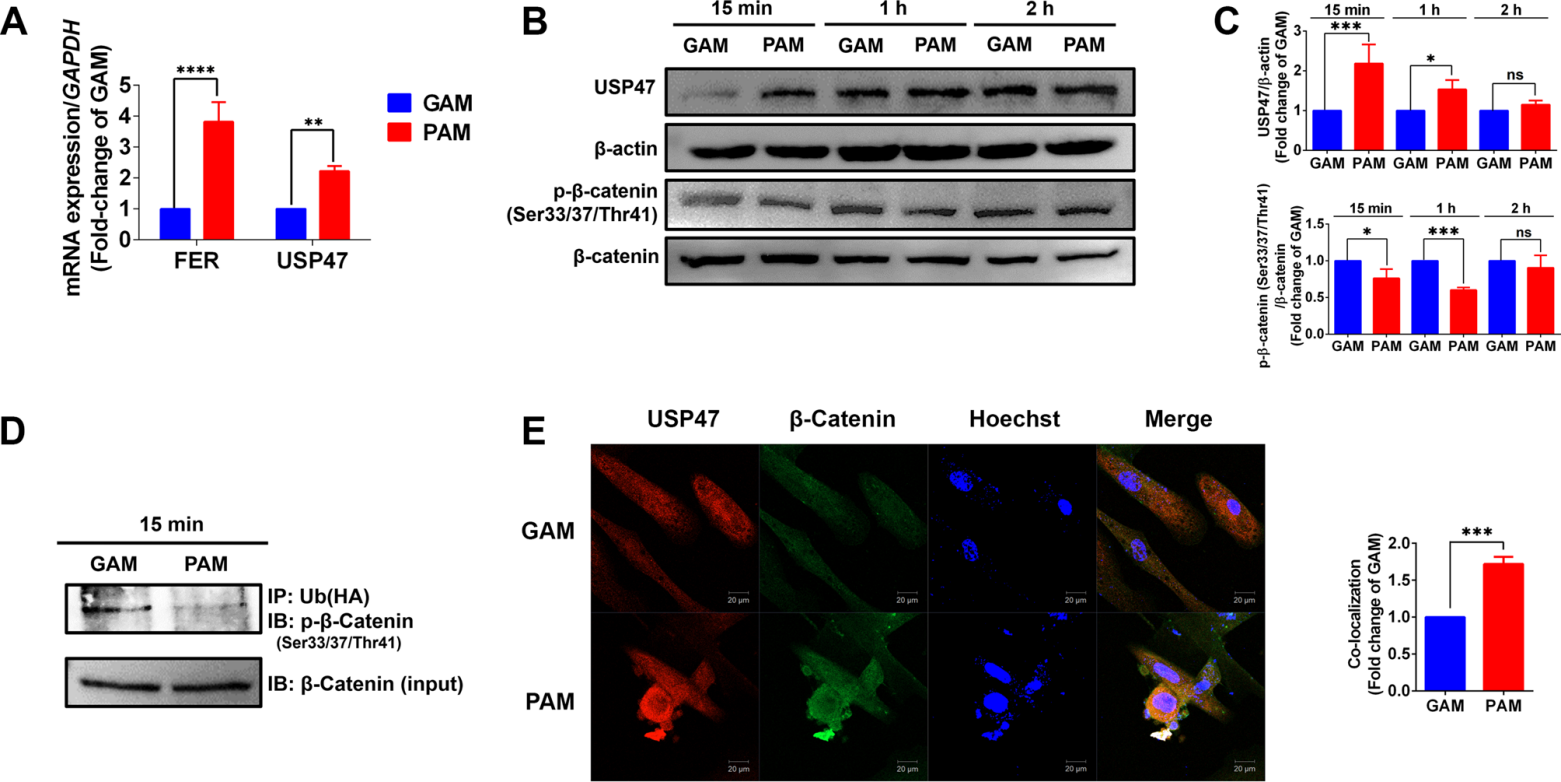
PAM impeded β-catenin degradation via USP47-regulated β-catenin ubiquitination in hDPCs. (**A**) The mRNA expressions of FER and USP47 genes in PAM-treated hDPCs, after 18 h of incubation. (**B**) Representative immunoblots and **(C)** Relative fold-change expressions of USP47 and p-β-catenin (Ser33/37/Thr41)/ β-catenin in PAM-treated hDPCs at 15 min, 1 h, and 2 h of incubation times. (**D**) The co-immunoprecipitation between p-β-catenin (Ser33/37/Thr41) and ubiquitin [Ub (HA)] in PAM-treated hDPCs at 15 min of incubation. (**E**) Representative images of USP47 and β-catenin immunofluorescence staining and quantitative analysis of USP47 and β-catenin co-localization in PAM-treated hDPCs compared to GAM control, after 15 min of incubation. The cells were observed under a confocal microscope with 20 × magnification. The scale bar indicates a 20 µm distance. All data are shown as the mean ± SD of experiments conducted in triplicate. Statistical significance; *P < 0.05; ***P < 0.001, and ****P < 0.0001. ns indicates not significant (*P* > 0.05).

We evaluated the transcriptome profiling of PAM-treated cells. HaCaT cells were treated with PAM or GAM and evaluated for gene expression profiling by RNA sequencing. From the RNA-seq analysis, we found that two genes involved in cell proliferation were highly expressed in PAM-treated conditions, namely FER and USP47 (unpublished data). Hence, we selected these genes to further investigate the expression levels in hDPCs by conducting qRT-PCR. Similarly, PAM significantly induced FER and USP47 expression, compared to GAM (Fig. 3A). Based on previous studies, USP47 impedes β-catenin ubiquitination and promotes cell proliferation^13^. Therefore, we further analyzed the protein expression of USP47 and phosphorylated β-catenin at Ser33/37/Thr41 position, which promotes β-catenin ubiquitination. The results revealed that PAM significantly increased USP47 expression and decreased β-catenin phosphorylation (Ser33/37/Thr41) at 15 min and 1 h after treatment (Fig. 3B and C). We confirmed the effect of PAM on inhibiting β-catenin ubiquitination by conducting co-immunoprecipitation between β-catenin and ubiquitin. Ubiquitinated β-catenin was decreased in PAM-treated cells (Fig. 3D). We investigated the correlation between USP47 and β-catenin by immunofluorescent imaging. The results showed that PAM significantly enhanced USP47 and β-catenin co-localization compared to GAM control (Fig. 3E). Thus, we concluded that PAM induces USP47 expression, which subsequently deubiquitinated β-catenin, leading to β-catenin stabilization in hDPCs.

### PAM increases INPP5D mRNA expression and induces Akt and GSK-3β phosphorylation in hDPCs

**Figure 4:**
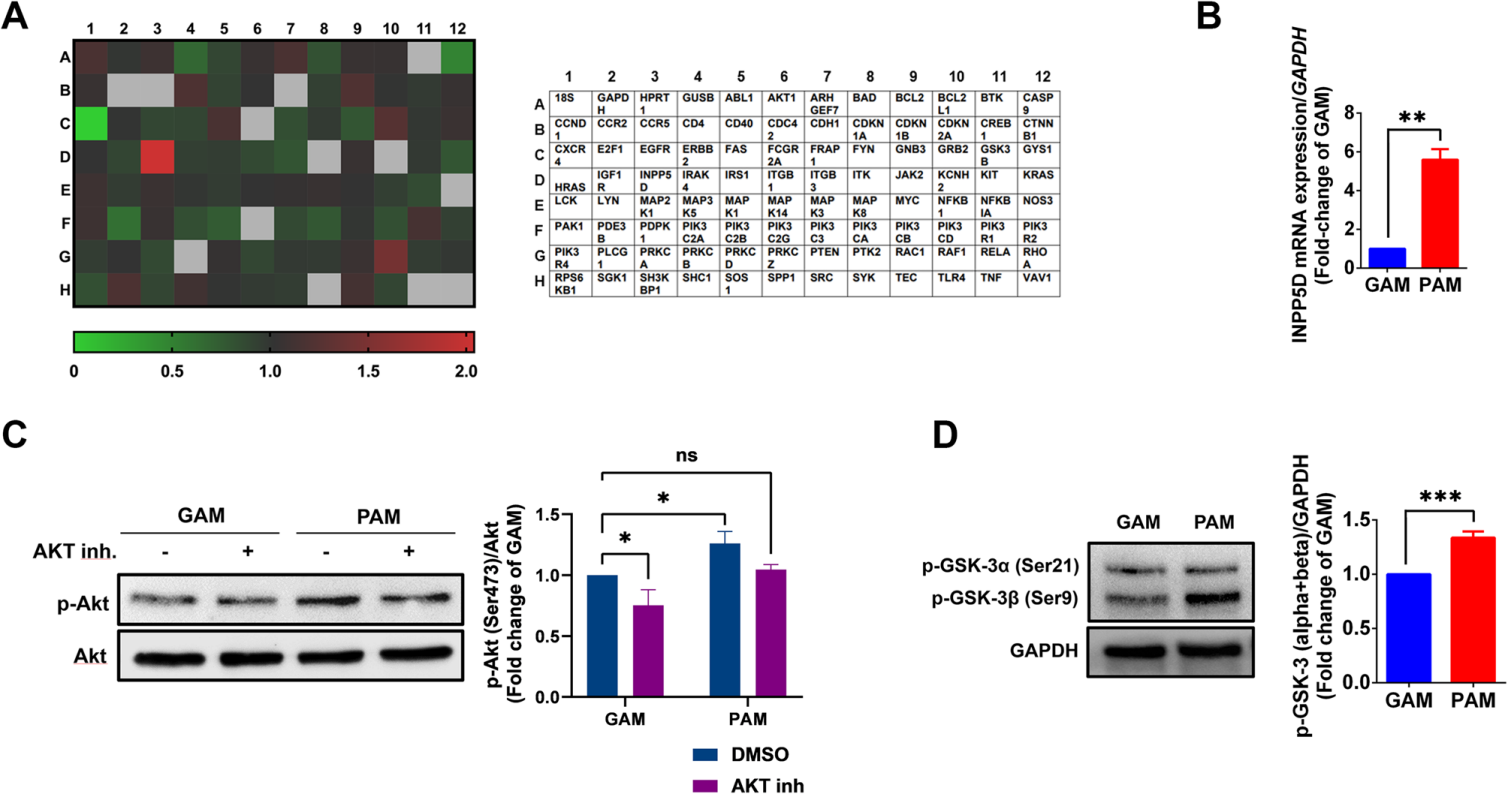
PAM increases INPP5D mRNA expression and induces Akt and GSK-3β phosphorylation in hDPCs. **(A)** The heatmap results of human PI3K signaling TaqMan™ mRNA array of PAM-treated hDPCs. (**B**) The mRNA expression of INPP5D gene in PAM-treated hDPCs. (**C**) Representative immunoblots and relative fold-change expressions of p-Akt (Ser473)/ Akt in GAM and PAM- treated hDPCs. Cells were pre-treated with Akt1/2 kinase inhibitor (5 µM) for 1 h, and then treated with GAM or PAM for 15 min. The cell lysates were subjected to Western blot analysis. (**D**) Representative immunoblots and relative fold-change expressions of p-GSK-3α/β (Ser21/9) in GAM and PAM-treated hDPCs. All data are shown as the mean ± SD of experiments conducted in triplicate. Statistical significance; *P < 0.05; **P < 0.01 and ***P < 0.001.

It has been shown that PI3K/Akt signaling pathway also plays a crucial role in cell proliferation and involves hair inductivity of DPCs and hair regeneration^20^. To determine the effect of PAM on inducing the PI3K/Akt signaling pathway, hDPCs were treated with PAM or GAM, and mRNA expression levels were measured using TaqMan™ Array Human PI3K Signaling. As shown in the heat map (Fig. 4A), the expression levels of 96 genes related to PI3K pathways were investigated and PAM highly induced INPP5D (a gene encoded SHIP1 protein) by two-fold compared to GAM control. We confirmed the result by conducting INPP5D qRT-PCR and found that INPP5D was significantly upregulated in the PAM-treated cells (Fig. 4B). Since SHIP1 is an upstream regulator of Akt signaling pathway^21^, we further evaluated protein expression of p-Akt and p-GSK-3β by western blot analysis. The results showed that PAM significantly induced Akt activity by increasing Akt phosphorylation at the Ser473 position and also enhanced GSK-3β phosphorylation at the Ser9 position, while PAM-pretreated with Akt inhibitor showed no significant difference, compared to GAM (Fig. 4C,D). These results suggested that PAM promotes hDPCs proliferation through upregulating INPP5D expression, and subsequently inducing Akt activity and suppressing GSK-3β function.

### PAM induces a core aggregate configuration of hDPCs with HaCaT keratinocytes

**Figure 5:**
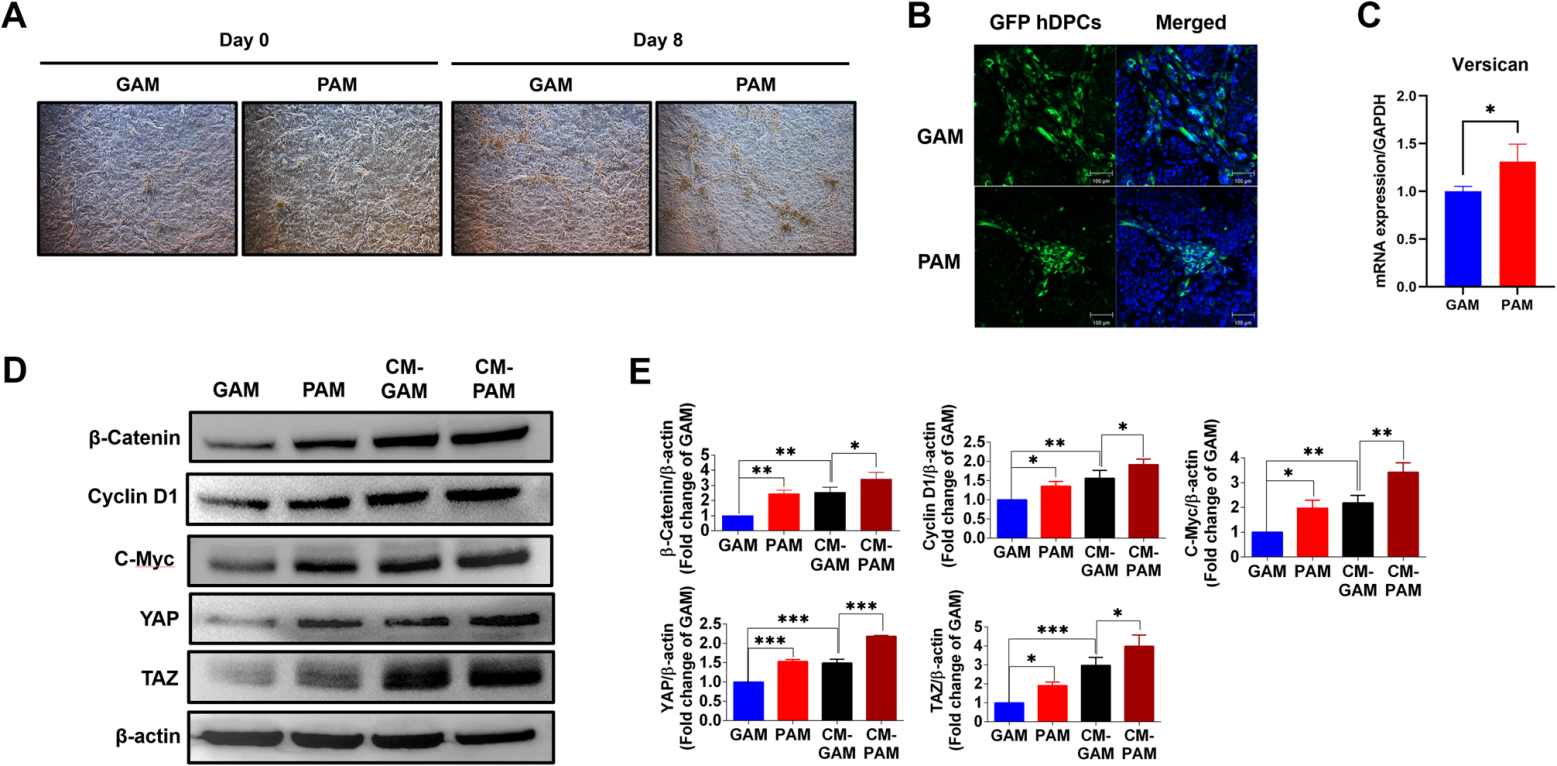
Conditioned media (CM) of hDPCs promotes protein expression of the β-catenin signaling pathway and YAP/TAZ in PAM-treated HaCaT cells. (**A**) The characteristics of hDPCs and HaCaT cells after 8 days of co-culturing and treating with PAM. The cell was visualized by Nikon Eclipse Ti-U inverted microscope with 4 × magnification. (**B**) The fluorescence images of GFP hDPCs and Hoechst 33,342 of the co-cultured PAM-treated hDPCs and HaCaT cells. The cells were observed under a confocal microscope with 10 × magnification. The scale bar indicates a 100 µm distance. (**C**) The mRNA expressions of versican genes in PAM-treated hDPCs, after 18 h of incubation. (**D**) Representative immunoblots and **(E)** Relative fold-change expressions of β-catenin, cyclin D1, c-Myc, YAP, and TAZ in HaCaT cells, after being treated with CM-derived hDPCs for 18 h. All data are shown as the mean ± SD of experiments conducted in triplicate. Statistical significance; * *P* < 0.05; ** *P* < 0.01, and ***P < 0.001.

It has been known that the aggregation of DPCs in hair bulbs is a crucial step for hair follicle induction^22^, and we found that PAM significantly induced hDPCs proliferation. To enhance DPCs aggregation, DPCs must come in contact with the surrounding environment, like keratinocytes. Co-cultures of DPCs with keratinocytes induce DPCs aggregation at the core surrounded by keratinocytes^23^. Hence, we examined the effect of PAM on enhancing hDPCs aggregation. HaCaT cells and hDPCs were co-cultured and treated with PAM or GAM. Cell morphology was visualized on days 0 and 8 of incubation. The results indicated that the aggregation of hDPCs could be observed on day 8, and the aggregated spots in the PAM-treated condition were more visible than in the GAM-treated control (Fig. 5A). To observe the aggregated profile, hDPCs were transfected with GFP construct before co-culturing. After treatment with PAM or GAM, the cells were stained with Hoechst 33342. hDPCs and HaCaT were rearranged to core‐shell configurations with hDPCs in the middle surrounded by HaCaT cells (Fig. 5B). To confirm the results, we next determined gene expression of versican; gene associated with DPCs aggregation^24^, by qRT-PCR in hDPCs. The result showed that PAM treatment significantly upregulates versican expression, compared to GAM (Fig. 5C). Therefore, we speculated that PAM induces hDPCs aggregation by triggering core‐shell configurations of hDPCs surrounded by keratinocytes.

### CM of hDPCs promotes protein expression of the β-catenin signaling pathway and YAP/TAZ in PAM-treated HaCaT cells

During hair follicle renewal, DPCs embedded in the hair bulb directly contact the surrounding keratinocytes to undergo asymmetric proliferation forming the new matrix pool^9^. The study also showed that growth factors secreted from CM of DPCs enhanced surrounding keratinocyte proliferation^25^. Hence, we investigated whether cultured PAM-treated HaCaT cells with CM from hDPCs could induce expression levels of cell proliferation-related proteins. HaCaT cells were treated with PAM or GAM and replaced with CM derived from hDPCs before evaluating the expression levels of β-catenin, cyclin D1, c-Myc, and YAP/TAZ. The results showed that CM-treated cells significantly increased the expression levels of these proteins as compared to HaCaT cells cultured with normal media. However, PAM-treated HaCaT cells with CM showed an enhancing effect on the induction levels of proteins compared to GAM-treated HaCaT cells with CM (Fig. 5D,E). Our finding suggested that PAM-treated HaCaT cells with CM derived from hDPCs synergistically enhance cell proliferation-related protein expression.

## Discussion

Alopecia, abnormal hair loss or baldness, is a disorder characterized by hair loss due to an interrupted hair production cycle. When a hair follicle is damaged, or this cycle is disrupted, hair may begin to fall out more quickly than regenerate, leading to symptoms such as hair falling out in patches or thinning. Some dogs, like humans, also exhibit hair loss when they have thickened skin, scaling, excessive shedding, genetic factors, and itching^26^. Thus, alopecia occurs both in people and dogs, and treatment options should be developed based on the mechanism of hair loss. Several therapeutic modalities are currently available to promote hair regrowth or prevent hair loss, including using 2% or 5% topical minoxidil for androgenic alopecia and anagen effluvium; triamcinolone acetonide for alopecia areata; and antifungal agents for tinea capitis. The signaling pathways, including a sonic hedgehog, Wnt/β-catenin, bone morphogenetic protein, transforming growth factor (TGF)-β, and notch signaling, have been implicated in the hair regeneration process. Among those, Wnt/β-catenin signaling has been well investigated.

The concept of the involvement of Wnt/β-catenin signals in follicular development stemmed from the β-catenin activation for cell proliferation, and the secreted Wnt proteins bind with the Frizzled receptor, thereby inactivating glycogen synthase kinase-3β (GSK-3β), which is an enzyme responsible for phosphorylation and ubiquitination-mediated degradation of β-catenin. Subsequent studies have reported that genetic ablation of the β-catenin gene in the epidermis leads to the failure of placode morphogenesis in mice^27^. In addition, studies indicated that the Wnt-dependent signaling pathway in the epidermis is the principle for folliculogenesis and increases the number of regenerated hair follicles^28,29^. Efforts are made to target these signaling pathways to design novel therapies for alopecia. Our results demonstrated that CAMP induces phospho-β-catenin (S552) protein expression and nuclear translocation to activate its target genes, thereby enhancing the effects on hair growth.

Moreover, CAMP also reduced the interaction between ubiquitin and phospho-β-catenin (Ser33/37/Thr41), diminishing β-catenin degradation. CAMP also increases mRNA and protein expression of USP47, a ubiquitin-specific proteinase that causes protein deubiquitination and then augments the protein level of β-catenin in the cells. Akt/PI3K signaling pathway is another important mechanism for hair maintenance and follicle regeneration^30^. The Akt/PI3K signaling assessed by the gene expression array demonstrated that INPP5D (SHIP1), a protein that is a main regulator on PI3K/Akt signaling, is the potential gene upregulated by CAMP treatment. The western blot data confirmed that CAMP upregulated p-Akt and p-GSK3β protein expression in hDPCs. The downstream protein of Akt/PI3K and GSK-3β plays a role in the Wnt/β-catenin signaling pathway as β-catenin does not enter the degradation pathway. CAMP activates β-catenin activation via several mechanisms, including USP47 and Akt/PI3K signaling proteins.

Besides Wnt/β-catenin and Akt/PI3K signaling, YAP and TAZ are the well-known crucial downstream proteins and transcription activators in the Hippo pathway, which regulates cell proliferation, renewal, and differentiation. The studies reported that the inhibition of transcription factor TEAD in human/mouse keratinocytes contributes to reducing cell proliferation and activating cell differentiation. The expression of YAP and TAZ in the basal stem/progenitor cell layer of the epidermis is required for skin homeostasis and cell proliferation in the mouse model^18^. YAP and TAZ also have been involved in hair growth, and YAP/TAZ activation increased cell growth and proliferation in dermal papillary cells^18^. This study demonstrated the induction of YAP and TAZ protein expression in hDPCs by CAMP treatment.

Moreover, the cell-cycle regulating markers, including c-Myc and cyclin D1, are also increased upon treating the cell with CAMP. The study reported that c-Myc was significantly expressed in the proliferative germinative epithelial cells, terminally differentiating matrix cells at the human follicle bulb, leading to hair fiber induction. c-Myc affects the follicles and plays a role in stem-transit amplifying cell switching, which is probably assumed as the site of human follicles stem cells^31^. Our study suggests that CAMP affected many beneficial signaling pathways that increase cell proliferation in hDPCs.

The study indicates that DPCs and the surrounding keratinocytes interact each other and conditioned media (CM) derived from DPCs contain growth factors that induces keratinocytes proliferation^25,32^. Hence, we investigated the effect of CM-derived from hDPCs and the enhancing effect of PAM and CM on increasing protein expression of β-catenin signaling in keratinocytes (HaCaT cells). The results showed that CM-GAM increases the protein expression of β-catenin signaling compared to GAM, while CM-PAM indicates the enhancing effect on stimulation of β-catenin signaling (Fig. 5D,E). This suggests that PAM contains long-lived radicals which trigger HaCaT signaling pathways. CM may contain growth factors to stimulate HaCaT cell growth. Although other molecular mechanisms may be needed to elucidate the factors produced by PAM-treated hDPCs, our data indicated that CAMP produced radicals and triggered signaling pathways to produce secreted proteins for cell proliferation.

For DPC aggregation analysis, the studies showed that DPCs become inductive and aggregate into the center of the keratinocytes to enhance the interaction between epithelium and mesenchyme during the anagen phase^33^. In this study, we found that PAM treatment can induce DPC aggregation, when co-cultured with keratinocytes. To elucidate the mechanism, we determine the mRNA expression of versican which plays a key role in maintaining DPCs induction during the anagen phase and promotes DPCs aggregation^33,34^. The result showed that PAM significantly upregulates versican expression, compared to GAM. Therefore, we concluded that PAM induces versican expression which subsequently promotes DPCs aggregation surrounded by keratinocytes.

**Figure 6:**
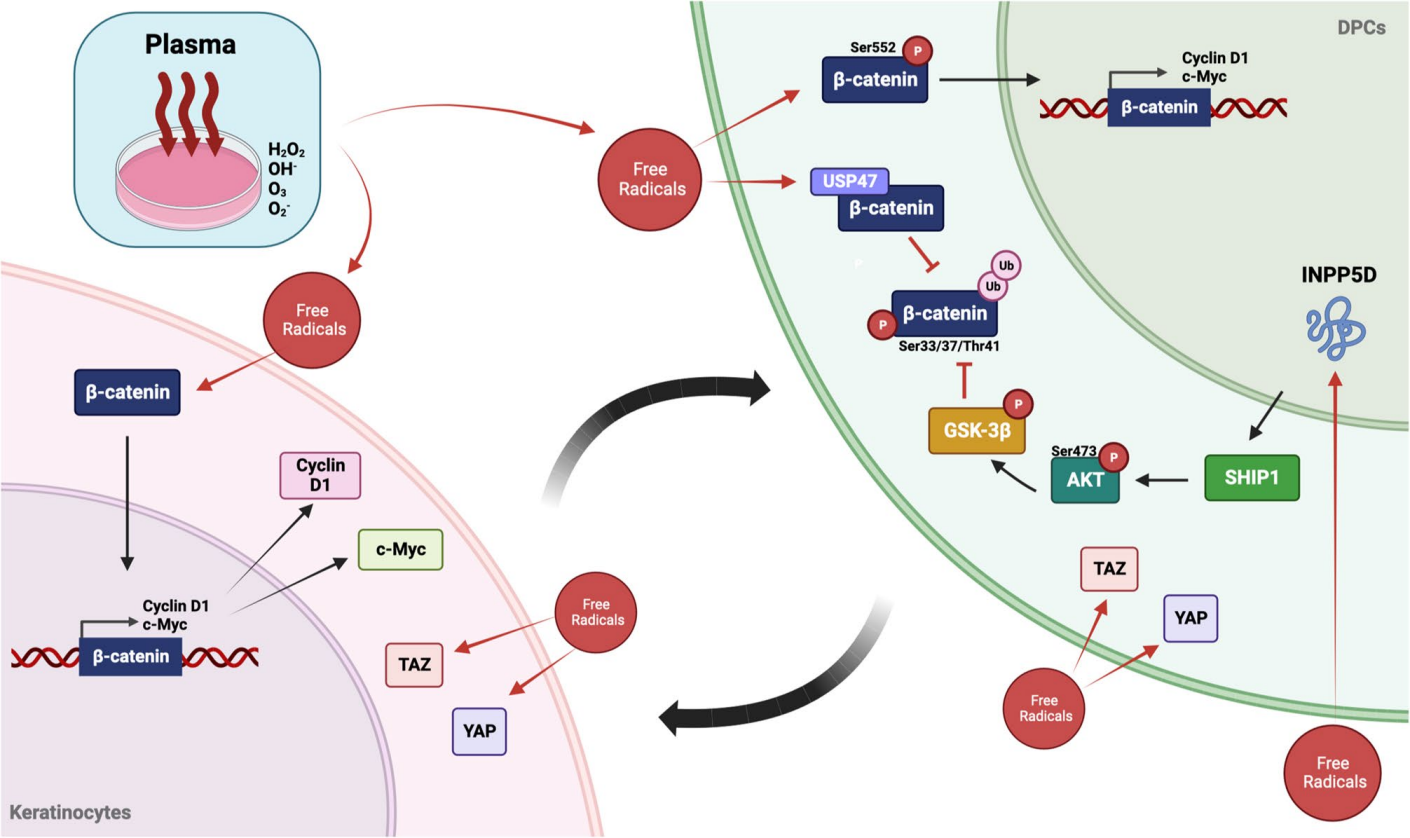
A schematic representation of the proposed molecular mechanisms of CAMP promoting hDPCs induction.

Despite the enormous effort in developing a safe and effective therapy for hair loss, there are few options available for clinical practice. The limited efficacy and notable side effect profiles of the existing therapies have led to extensive research on understanding hair biology, especially the molecular mechanisms underlying hair loss and the hair follicle regeneration process. Thus, efforts have been made to develop therapies for hair loss treatment by targeting signaling pathways. Compared to other types of plasma, the CAMP has a higher electron temperature than plasma obtained by the indirect and high-frequency discharge. Due to the high temperature of the electrons, there is also a higher dissociation and ionization degree, which is reflected in a wide range of uses of this type of discharge, including wound healing^35^, surface decontamination of fruits^36^, or deposition of thin layers^37^. Our finding suggested that CAMP has a therapeutic effect on stimulating hair regrowth through Akt/β-catenin signaling, YAP/TAZ Hippo pathway, and USP47-associated β-catenin deubiquitination in hDPCs (Fig. 6). We also found that CAMP enhances hDPCs aggregation by inducing core‐shell configurations of hDPCs surrounded by keratinocytes. CM derived from hDPCs exerts an enhancing effect on enhancing β-catenin signaling and YAP/TAZ Hippo pathway in HaCaT keratinocytes. CAMP could serve as a novel candidate for hair loss treatment.

## Materials and methods

### Chemicals and reagents

Dulbecco's Modified Eagle Medium (DMEM), Fetal bovine serum (FBS), phosphate-buffered saline (PBS), Penicillin–Streptomycin, SuperSignal™ West Femto Maximum Sensitivity Substrate, and Pierce™ BCA Protein Assay Kit were purchased from Thermo Scientific (Logan, UT, USA). 3-(4,5-Dimethylthiazol-2-yl)-2,5-diphenyltetrazolium bromide (MTT) and Akt1/2 kinase inhibitor (Cat. #A6730) was purchased from Sigma-Aldrich (St. Louis, MO, USA). RIPA lysis buffer was purchased from Biomax, Seoul, Korea. The fluorescence mounting medium (S3023) was purchased from Agilent, CA, USA. Mouse anti-β-catenin (Cat. #610,154) was purchased from BD Biosciences, Franklin Lakes, USA. Rabbit anti-cyclin D1 (Cat. #92G2), rabbit anti-c-Myc (Cat. #5605P), rabbit anti-YAP/TAZ (D24F4) (Cat. #8418S), rabbit anti-p-β-catenin (S552) (Cat. #9566P), rabbit anti-p-β-catenin (S33/37/T41) (Cat. #9561P), rabbit anti-p-Akt (S473) (Cat. #9271), rabbit anti-Akt (Cat. #9272), p-GSK-3α/β (Ser21/9) (Cat. #9331) were purchased from Cell Signaling Technology (Danvers, MA, USA). Mouse anti-USP47 (Cat. #SC-100633), mouse anti-HA tag (F-7) (Cat. #SC-7392), mouse anti-β-actin (C4) HRP (Cat. #SC47778), HRP-conjugated anti-GAPDH antibody (G-9) (Cat. #SC-365062 HRP) were purchased from Santa Cruz Biotechnology (Dallas, TX, USA). Goat anti-rabbit IgG (H + L) Secondary antibody HRP conjugated (Cat. #31,460) and Rabbit anti-Mouse IgG (H + L) Secondary antibody HRP conjugated (Cat. #31,450) were purchased from Invitrogen (Rockford, USA).

### Cell culture

Human hair DPCs (hDPCs) were purchased from ScienCell (Cat. #2400, CA, USA). Cells were cultured in mesenchymal stem cell medium (Cat. #7501, ScienCell) containing 10% FBS, 1% mesenchymal stem cell growth supplement (MSCGS, Cat. No. 7552, ScienCell), and 1% penicillin–streptomycin. The immortalized human keratinocyte (HaCaT) cells were purchased from Cell Line Service (Eppelhein, Germany). Cells were cultured in high glucose DMEM containing 10% FBS and 1% penicillin–streptomycin. Both cells were cultured and maintained in a 5% CO_2_ humidified incubator at 37 °C.

### Plasma device

A CAMP device (Bio Stimulation Microwave Plasma v1.0, Ion Medical Inc.) was used for producing plasma in this research (Fig. 1B). The energy is an ionized gas that generates controllable radicals, including short-lived radicals and long-lived radicals. The device ignited the microwave plasma at an inner electrode in the glass tube. The energy was delivered through the cylinder to the target sites by an argon carrier gas. The CAMP condition could be altered to 30W, 35W, and 40W, depending on the power consumption and gas velocity (10–20 L/min). The CAMP was set at 30W and gas velocity at 10 L/min in this experiment. The exposing temperature was maintained below 38 °C.

### Plasma-activating media (PAM)-treated cells

DMEM containing 10% FBS and 1% penicillin–streptomycin was exposed to various CAMP doses and treated in the seeded cells. PAM-treated cells were incubated at 37 °C for 15 min, and then the PAM was removed. Cells were replaced with fresh media and further incubated for 18 h before harvesting. For the gas control condition, cells were treated with argon gas-activated media (GAM) and were incubated with the same condition as above (Fig. 1B).

### MTT assay

The hDPCs were seeded in 96-well plates at a density of 3 × 10^3^ cells/well, and then the cells were treated with gas (30W, 35W, or 40W) for 40 s or PAM (30W, 35W, or 40W) for 10, 20, or 40 s. After 18 h incubation, cell viability was investigated by adding an MTT reagent and further incubated for 3 h. The purple formazan precipitate was dissolved by adding 10% SDS and incubated overnight at 37 °C. The absorbance was measured by a microplate reader at 570 nm (Thermo Fisher Scientific, Waltham, MA, USA).

### Quantitative RT-PCR

hDPCs were seeded in 6-well plates at a density of 1 × 10^5^ cells/well, and then the cells were treated with GAM or PAM at 30W for 10 s. After incubation for 18 h, RNA was extracted using Trizol reagent (Ambion, CA, USA) and was converted to cDNA using Verso cDNA Synthesis Kit (Thermo Scientific, UT, USA). The quantitative RT-PCR was performed using Applied Biosystems™ SYBR Green Master Mixes (Thermo Scientific, UT, USA) and analyzed by QuantStudio 1 Real-Time PCR System (Thermo Fisher Scientific, UT, USA). The lists of primers are indicated below.

$$\begin{array}{ll}\text{FER:}&\text{FW 5}'\text{ TGA AGA GCA GAC CCG TTT GG 3}'.\\ & \text{RW 5}'\text{ AGC GTG TCC ATG ATG AGG TG 3}'.\\\text{USP47:}& \text{FW 5}'\text{ GGC TTC TAC TAG GTG GCG TC 3}'.\\ & \text{RW 5}'\text{ TCA CCA TCA CTT CTC CAG GT 3}'.\\ \text{INPP5D:}& \text{FW 5}' \text{ GGG AGA AAG TCC TCC GAC AC 3}'\\&\text{RW 5}'\text{ CAA ACA TCT CGG GCT TCG TC 3}' \\ \text{Versican:} &\text{FW 5}'\text{ TGT GTT TCA CTA CAG GGC GG 3}'.\\ & \text{RW 5}'\text{ GCG TCA CAC TGC TCA AAT CC 3}'\\ \text{LEF1}& \text{FW 5}' \text{ TCCCGTGAAGAGCAGGCTAAAT 3}'\\& \text{RW 5}'\text{ TTGTCTCTTGCAGACCAGCCT 3}' \\ \text{GAPDH:} &\text{FW 5}'\text{ GAC CAC AGT CCA TGC CAT CAC T 3}'\\ & \text{RW 5}'\text{ TCC ACC ACC CTG TTG CTG TAG 3}'\end{array}$$

### Gene expression array

The hDPCs were seeded and treated with the same conditions as quantitative RT-PCR. After RNA extraction, cDNA synthesis and reaction preparation were conducted, followed by the protocol from TaqMan™ Array Human PI3K Signaling (Cat. #4,414,172, Applied Biosystems™, Waltham, USA). Briefly, the cDNA sample (50 ng per 20 µL of reaction) was mixed with 2 × TaqMan® Fast Advanced Master Mix (Applied Biosystems™, Waltham, USA) to obtain the final volume of 2,160 µL. The cDNA‑Master Mix was added to the 96‑well Standard (0.2 mL) TaqMan® Array Plates at 20 µL per well. The plates were sealed with microplate sealing film (Thermo Scientific, UT, USA). The experiments were set up following the protocol and analyzed by QuantStudio 1 Real-Time PCR System.

### Western blot analysis

The hDPCs were seeded in 60-mm dishes at a density of 2 × 10^5^ cells/well, and then the cells were treated with GAM or PAM at 30W for 10 s. For p-β-catenin and USP47 analysis, cells were further incubated in the replaced media for 15 min and 1 and 2 h after GAM or PAM removal, respectively. After incubation, cell proteins were extracted with RIPA buffer, including a protease inhibitor. The proteins were sonicated and centrifuged at 14,000 rpm, 20 min, and 4 °C. The supernatants were collected, and the proteins were measured by Pierce™ BCA Protein Assay Kit (Thermo Scientific, UT, USA). The proteins were equalized with 5 × SDS-PAGE diluted in RIPA buffer and heated at 98 °C for 5 min. Then, 5% stacking and 8% or 10% separating acrylamide gel were used for electrophoresis, and polyvinylidene fluoride (PVDF) membranes (0.2 μm) were used for protein transfer. The membranes were blocked with 5% non-fat dry milk in TBST (0.1% Tween 20) for 30 min before being incubated with primary antibodies overnight on the shaker at 4 °C. Goat anti-Mouse/Rabbit IgG (H + L) antibodies (dilution 1:2,000) (Invitrogen, Waltham, USA) were used as secondary antibodies. Supersignal West Femto maximum sensitivity substrate (Thermo Scientific, UT, USA) was used for visualization. The membranes were detected by Alliance Q9 advanced chemiluminescence imager (UVITEC, Cambridge, UK), and the band intensity was calculated by Image J software (Version 1.53).

### Immunoprecipitation

The hDPCs were seeded in 10-cm cell culture dishes at a density of 8 × 10^5^ cells/dish. Cells were then transfected with HA tag-ubiquitin (Ub-HA) plasmids using PolyJet reagent according to the following protocol (Cat. #SL100688, SignaGen Laboratories, Frederick, MD, USA). Cells were treated with GAM or PAM at a low dose for 10 s and incubated for 15 min before harvesting protein. The proteins (0.5 mg) were incubated with HA-Tag mouse mAb or normal mouse IgG by rotating overnight at 4 °C. Then, the complexes were pulled down by adding Pierce™ Protein A/G Magnetic Beads (Cat. #88,802, Thermo Scientific, UT, USA). After incubation, the bead complexes were collected and washed with 0.05% TBST. The protein complexes were eluted by adding 1 × SDS-PAGE reducing buffer and rotated for 20 min at RT. The expression levels were determined by western blot analysis, and the membrane was probed with rabbit anti-p-β-catenin (S33/37/T41) for IP conditions and mouse anti-β-catenin for input conditions.

### Immunofluorescence

The hDPCs were seeded in PDL-coated cover slip placed on 6-well plates at a density of 1 × 10^5^ cells/well. The cells were then treated with GAM or PAM for 10 s and further incubated for 15 min after replacing with fresh culture media. Cells were washed and fixed with cold PBS and 4% PFA, respectively. Cells were permeabilized and blocked using 0.3% Triton X-100 and 5% BSA, respectively. Cells were stained with rabbit anti-β-catenin (1:200) and mouse anti-USP47 (1:200) antibodies overnight at 4 °C. After washing, cells were stained with fluorescein goat anti-rabbit (H + L) (Cat. #F2765, Invitrogen, Waltham, USA) and Alexa Fluor 568 goat anti-mouse (H + L) (Cat. #A11004, Invitrogen, Waltham, USA) for 1 h at RT. Hoechst 33,342 (10 µg/mL) was used for nuclei staining. Cells were mounted on slides with anti-fade and sealed with a mounting medium. Cells were visualized under a confocal microscope, and the data were analyzed by Image J software.

### Co-culture and CM preparation

For co-culture preparation, HaCaT and hDPCs were mixed in the same tube at 1 × 10^5^ cells/well density and seeded in 35-mm dishes. The cells were treated with GAM or PAM for 10 s. The cells were visualized on day 0 and day 8 by Nikon Eclipse Ti-U inverted microscope. For monitoring cell location, hDPCs were transfected with pmEGFP-C1 (Cat. #36412, Addgene, Teddington, UK) using PolyJet™ reagent. The cells were trypsinized and co-cultured with HaCaT cells. The co-cultured cells were treated as above. After incubation, both cells were stained with Hoechst 33342 (10 µg/mL) for 10 min at 37 °C. The cells were mounted on slides and visualized under a confocal microscope.

For CM preparation, hDPCs and HaCaT cells were separately seeded in 60-mm dishes at a density of 1 × 10^6^ cells/well, and then the cells were treated with GAM or PAM at 30W for 10 s. After incubation, cells were replaced with CM derived from hDPCs and further incubated for 18 h before harvesting.

### Statistical analysis

The results were indicated as means ± standard error (SE) with three independent experiments. The data were analyzed by Student's t-test and one-way analysis of variance followed by post hoc Dunnett test using GraphPad Prism 9 software. The p-values less than 0.05 were indicated as statistically significant.

## Data Availability

All data generated or analyzed during this study are included in this published article.
